# A comparison of different methods to maximise signal extraction when using central venous pressure to optimise atrioventricular delay after cardiac surgery

**DOI:** 10.1016/j.ijcha.2024.101382

**Published:** 2024-03-12

**Authors:** Ioana Cretu, Alexander Tindale, Maysam Abbod, Wamadeva Balachandran, Ashraf W. Khir, Hongying Meng

**Affiliations:** a Brunel University London, London, UK; b Royal Brompton and Harefield Hospitals, Guy’s and St Thomas’ NHS Foundation Trust, London, UK; c Durham University, Durham, UK

**Keywords:** Atrioventricular delay, CRT, CVP, Filtering, Optimisation, Temporary pacing

## Abstract

**Objective:**

Our group has shown that central venous pressure (CVP) can optimise atrioventricular (AV) delay in temporary pacing (TP) after cardiac surgery. However, the signal-to-noise ratio (SNR) is influenced both by the methods used to mitigate the pressure effects of respiration and the number of heartbeats analysed. This paper systematically studies the effect of different analysis methods on SNR to maximise the accuracy of this technique.

**Methods:**

We optimised AV delay in 16 patients with TP after cardiac surgery. Transitioning rapidly and repeatedly from a reference AV delay to different tested AV delays, we measured pressure differences before and after each transition. We analysed the resultant signals in different ways with the aim of maximising the SNR: (1) adjusting averaging window location (around versus after transition), (2) modifying window length (heartbeats analysed), and (3) applying different signal filtering methods to correct respiratory artefact.

**Results:**

(1) The SNR was 27 % higher for averaging windows around the transition versus post-transition windows. (2) The optimal window length for CVP analysis was two respiratory cycle lengths versus one respiratory cycle length for optimising SNR for arterial blood pressure (ABP) signals. (3) Filtering with discrete wavelet transform improved SNR by 62 % for CVP measurements. When applying the optimal window length and filtering techniques, the correlation between ABP and CVP peak optima exceeded that of a single cycle length (R = 0.71 vs. R = 0.50, p < 0.001).

**Conclusion:**

We demonstrated that utilising a specific set of techniques maximises the signal-to-noise ratio and hence the utility of this technique.

## 1 Introduction

Optimisation of atrioventricular (AV) delay can result in improved haemodynamics in patients after surgery [1] and in stable outpatients with cardiac resynchronization therapy (CRT) [2], [3], [4].

A commonly-used and well-validated approach is the assessment of blood pressure changes that occur during alternations between a reference AV delay (usually 120 ms) and each tested AV delay [4], [5], [6]. The changes around each transition allow an AV delay optimisation curve to be drawn and the optimal AV delay to be calculated. These studies also show that different techniques used in processing and analysing this data can have significant impacts on the results.

Recent work from our group has shown a strong inverse relationship between central venous pressure (CVP) and arterial blood pressure (ABP) when examining AV delay changes for temporary pacemakers after cardiac surgery [1]. This finding is particularly relevant as CVP, an accessible parameter through pacemaker leads in central veins, can be directly measured, offering a practical advantage for real-time monitoring and optimisation. Unlike arterial pressure sensing, which lacks implantable devices for real-time monitoring, CVP sensing stands out as a feasible alternative. CVP is not only a critical marker routinely monitored in patients recovering from cardiac surgery but also serves as both a dependent and independent indicator of cardiac output, positioning it as an essential target for optimising patient outcomes. However, there is a possibility that large changes in CVP during the respiratory cycle may outweigh some of the signal from this optimisation methods, and therefore there is a need to explore techniques to maximise the signal-to-noise ratio (SNR).

Previous work has looked at maximising SNR of arterial signals but not central venous signals in this setting. When analysing AV delay using the approach around transitions, the blood pressure is averaged for a certain number of beats before and after each transition to ascertain the relative effect of each tested AV delay versus the reference AV delay. Previous research has assessed both changing the length of this averaging window (i.e. the number of heartbeats analysed) and the position of the window (i.e. whether it should start immediately after the transition or a number of beats either side) [6], [7], [8].

These results suggest that the most signal is in the data immediately after the transition, where the cardiac output has changed but before the patient's homeostatic mechanisms (such as vasodilation or vasoconstriction) are activated to return blood pressure to the pre-transition state [5].

Therefore, we set out to answer three key questions pertaining to maximising the signal-to-noise ratio of CVP analysis during temporary pacing optimisation:

(1) Is the SNR higher when analysing around the transition or only after the transition? If the main effect of optimisation is seen immediately after a transition, the analysis after transition points could offer the bulk of the signal with reduced noise and processing requirements.
(2) Can window length be optimised, especially in relation to the respiratory cycle, to maximise the signal-to-noise ratio? CVP fluctuates greatly with respiration [9] and therefore aligning the averaging window to multiples of respiratory cycle lengths may be a good method of maximising signal-to-noise ratio, as has been shown with arterial signals [6].
(3) Similarly, can different filtering strategies be used to offset respiratory artefact? To answer this, we systematically compare two filtering techniques: Discrete Wavelet Transform (DWT) and Asymmetric Least Squares (ALS) across 1 to 20 heartbeats around each transition.

Therefore, the overall aim is to describe a number of different strategies that can be used to maximise signal-to-noise ratios for CVP and ABP measurements in patients after cardiac surgery. The predominant focus is on the venous system because it is a potential optimisation target for implantable devices, which have leads directly in the great veins.

## 2 Methods

### 2.1 Subjects

Sixteen patients with dual-chamber temporary cardiac pacing devices were studied within 72 h following cardiac surgery. To be eligible for the study, participants were required to be over 18 years of age, capable of giving informed consent, possess any degree of left ventricular function, and have undergone open cardiac surgery. This included Coronary Artery Bypass Grafting (CABG), aortic, mitral, and tricuspid valve surgeries, or combinations thereof, necessitating the placement of temporary epicardial wires. Patients with a pre-existing permanent pacemaker were excluded. Of the sixteen participants, nine underwent CABG, three had aortic valve replacement (AVR) and root replacement, two received AVR alone, one underwent tricuspid valve (TV) replacement, and one had CABG combined with mitral valve repair. The age range of the participants was 41–80 years, with a mean age of 71 years. Among them, four were female, and twelve were male. Fourteen patients had an underlying sinus rhythm, while two were pacing-dependent.

### 2.2 Data collection

Invasive arterial blood pressure (ABP) was transduced from the right radial artery and the right superior vena cava (CVP) using Edwards Lifesciences TruWave pressure transducers. ECG signals were taken using a Boston Scientific Labsystem Pro electrophysiology recording system. Digital to analog conversion occurred with a National Instruments DAQ card and LabVIEW software (National Instruments, TX, USA).

### 2.3 Measurement of relative blood pressure changes for different AV delays

Beat-to-beat blood pressure was continuously recorded while pacing in DDD mode (dual chamber pacing, sensing, inhibition and stimulation) at the lower rate of 90 beats per minute (bpm) or 10 bpm above sinus rhythm. All patients began pacing at the reference AV delay of 120 ms before transitioning rapidly to a tested AV delay, which ranged from 40 ms to 280 ms in 40 ms increments for 20 beats, before transitioning back to the reference AV delay. This transition process occurred 8 times for each tested AV delay. Testing was stopped when intrinsic conduction occurred or the tested AV delay reached 280 ms. At the end we obtain a mean change in blood pressure (one for ABP and one for CVP) for each tested AV delay. The precise position of the averaging window (around the transition, and posttransition only), and the duration of the averaging window (number of beats and respiratory cycle length) was varied as part of the experimentation process.

Throughout this manuscript, when we refer to ABP values, we are referring to peak (or systolic) arterial blood pressure.

### 2.4 Ethics

All patients gave written, informed consent. This study was approved by the South West – Cornwall and Plymouth Research Ethics Committee as part of the PACESIM trial (ISRCTN15383573).

### 2.5 Measurement of signal-to-noise ratio

Signal-to-noise ratio (SNR) it is a simple and effective way of measuring the efficiency of the optimisation technique, and it is calculated using the same approach conducted in previous studies [4], [6], which defined SNR as the ratio between the range of values obtained for different AV delay settings (difference between the maximum and minimum changes in systolic blood pressure) and the mean standard error of the pressure measurements at each AV delay setting.

### 2.6 Position of the averaging window

**Fig. A1:**
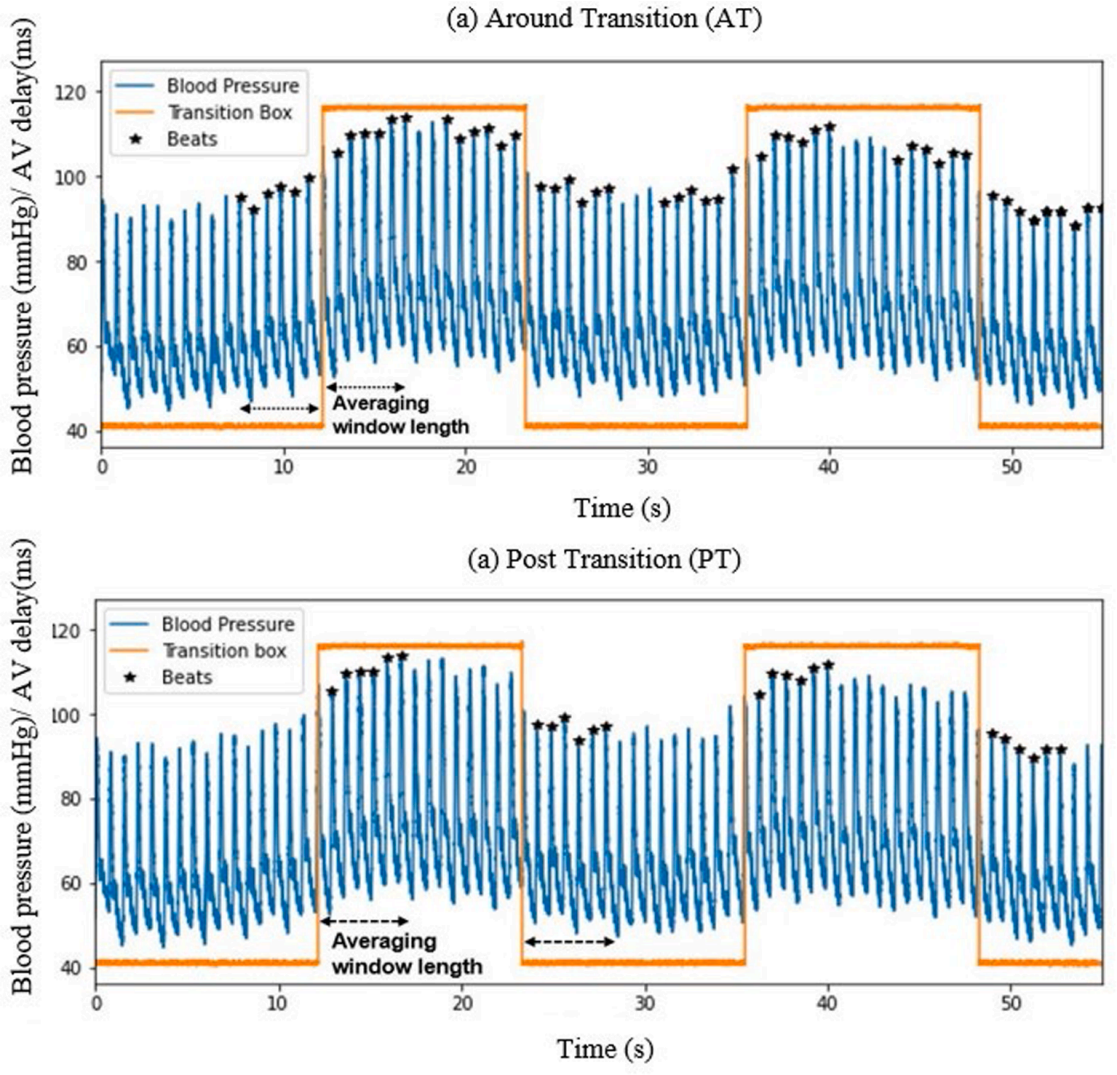
Example of around-transition (AT) and post-transition (PT) window selection for a tested AV delay of 40 ms.

In order to identify the most efficient location for data selection, we compared the SNR of analysing beats directly around the transition (AT) with those only taken post-transition (PT) (Appendix Fig. A1). Both methods were tested analysing the mean difference in pressure for an averaging window between 1 and 20 beats for each tested AV delay compared to the reference AV delay. The same process was performed but where the number of beats varied as a proportion of each patient's personalised respiratory cycle length, ranging from 0.25 respiratory cycles to 2 cycles. In order to establish the best position of the averaging window we compared the mean SNR across all patients for each of the described methods.

### 2.7 Noise correction of the central venous pressure and arterial line blood pressure signals

**Fig. 1:**
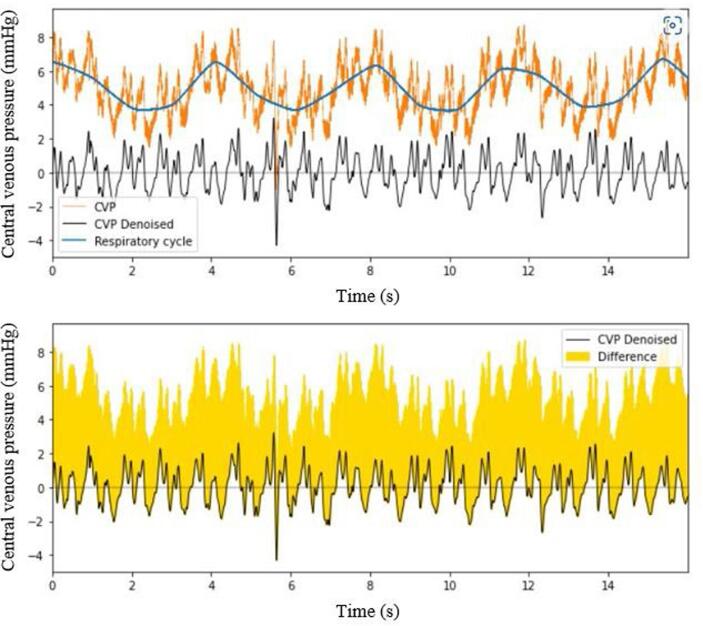
The result of filtering the central venous pressure (CVP) signal. The unfiltered central venous pressure (CVP) signal is shown in orange. The respiratory effect as calculated by asymmetric least squares smoothing (ALS) is shown in blue. This respiratory effect is then subtracted from the original unfiltered signal to obtain the filtered signal, shown at the bottom of the figure in black. The difference between the original signal and the filtered CVP signal is shown in the bottom panel in yellow. (For interpretation of the references to colour in this figure legend, the reader is referred to the web version of this article.)

Central venous pressure fluctuates to a large degree during respiration, as shown in Fig. 1. Although not as pronounced, the ABP signals are also affected by changes in intrathoracic pressure. Therefore, we investigated the efficacy of two different methods of baseline filtering on both CVP and ABP signals: Asymmetric Least Squares Smoothing (ALS) [10], and Discrete Wavelet Transform (DTW) [11]. In both cases, the baseline is substracted from the original signal, but also used as the respiratory trace in our analysis.

In order to determine the best form of filtering for all signal lengths in this dataset, we evaluated the filtering in two ways. The primary methods was to calculate the SNR for different combinations of filtered and unfiltered signals as an average of window lengths from 1 to 20 beats. The second method was to compare the correlation of individual data points (i.e. the change from reference for each AV delay for each patient and for each different averaging window length) as a combination of different methods of filtering. For each of DWT and ALS-filtered signals there are 4 possible combinations to compare: (1) CVP unfiltered (CVPU) and ABP unfiltered (ABPU), (2) CVP filtered (CVPF) and ABP unfiltered, (3) CVP unfiltered and ABP filtered (ABPF), and (4) both CVP and ABP filtered. The correlation between the gold standard (ABP) and CVP was then compared via the strength of this relationship.

### 2.8 Averaging window length

In order to determine the optimal averaging window length, we performed two experiments. In the first experiment we set the window length to be a fixed number of beats. We started from a window length of one beat and progressively increased it up to 20 beats. In the second experiment, we adjusted the window length according to each individual's respiratory rate. We did this by using the respiratory trace extracted using the filtering methods and calculating the number of heartbeats per breath for that particular patient. Then, different proportions of the respiratory cycle ranging from half respiratory cycle to 2 respiratory cycles were tested and compared using the SNR.

We also compared the utility of peak and mean values of CVP for analysis, where a single peak was taken for ABP and two peaks (corresponding to the a and v waves) were taken from CVP signals.

### 2.9 Statistical analysis

The data processing and automatic result extraction were conducted using a custom-built software developed in Python 3.7. For statistical analysis, specifically to compute the correlation between measurements through the Wilcoxon Signed-Rank Test, IBM SPSS Statistics 29.0.1.0 was employed. Furthermore, the visualization of data, including all generated plots, was facilitated using GraphPad Prism version 9.1.2.226.

## 3 Results

### 3.1 Optimal location of the averaging window

**Fig. A2:**
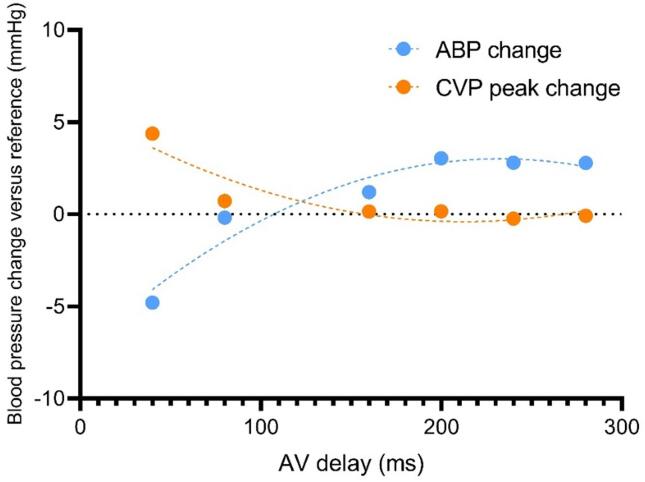
The impact of the AV delay change on the filtered central venous pressure (blue) and arterial line blood pressure (orange).

Choosing an averaging window AT led to higher SNR than using windows only after the transition (PT). This finding held both for heartbeat number-based and respiratory cycle-based window lengths (Appendix Fig. A2).

When correcting the ABP signals with ALS filtering there was a 17 % reduction (P < 0.001) using a post-transition window compared to around-transition for all beat lengths and 19 % (P = 0.02) when adjusting the window length to the respiratory cycle.

For peak CVP measurements, the SNR showed a similar pattern. Post-transition windows resulted in a lower SNR than around-transition windows: 21 % lower (P < 0.001) for fixed-beat windows and 13 % lower (P = 0.06) for respiratory cycle-based windows. The location of the averaging window also affected the measurements of mean CVP signal, with a drop in SNR of 16 %(P < 0.001) and 12 %(P = 0.05) for beat and respiratory cycle window sizes, respectively. These observations were consistent across both ALS and DWT filtering techniques.

**Fig. A3:**
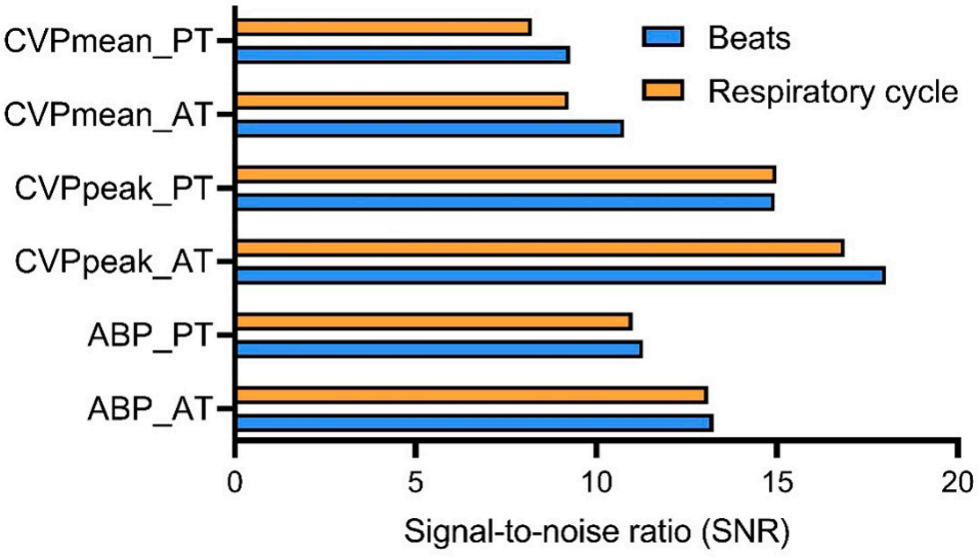
SNR for different methods of averaging window location and length selection. The results are an average across all beats and all respiratory cycle proportions for all patients when both CVP and ABP signals were filtered using ALS filtering.

In terms of correlation between SBP and CVP signals, we observed that around-transition window selections also resulted in higher correlation coefficients than post-transition window selections for both filtering methods (Table 1). These results are also shown graphically in Appendix Fig. A3.

**Table 1:** The correlation coefficient (R) and the statistical significance (P-value) between CVP and ABP signals using different methods of CVP measurement (CVP peak and CVP mean). The data presented is a mean across all patients for a window length of 5 heartbeats.

Location	Around Transition				Post Transition			
Filtering	CVP peak		CVP mean		CVP peak		CVP mean	
	R	P	R	P	R	P	R	P
*Asymmetric Least Square Smoothing*								
CVP filtered								
ABP unfiltered	−0.56	<0.001	0.37	<0.001	−0.40	<0.001	0.46	<0.001

*CVP unfiltered*								
ABP unfiltered	−0.58	<0.001	0.28	0.01	−0.40	<0.001	0.38	<0.001

*CVP unfiltered*								
ABP filtered	−0.52	<0.001	0.26	0.02	−0.36	<0.001	0.40	<0.001

*CVP filtered*								
ABP filtered	−0.50	<0.001	0.39	<0.001	−0.38	<0.001	0.48	<0.001

*Discrete Wavelet Transform*								
CVP filtered	−0.59	<0.001	0.46	<0.001	−0.46	<0.001	0.48	<0.001
ABP unfiltered								
CVP unfiltered	−0.58	<0.001	0.28	0.01	−0.40	<0.001	0.38	<0.001
ABP unfiltered								
CVP unfiltered	−0.24	0.03	0.27	0.02	−0.36	<0.001	0.47	<0.001
ABP filtered								
CVP filtered	−0.27	0.01	0.31	0.005	−0.41	<0.001	0.52	<0.001
ABP filtered								

### 3.2 Effect of noise correction on the CVP and ABP signals

**Fig. A4:**
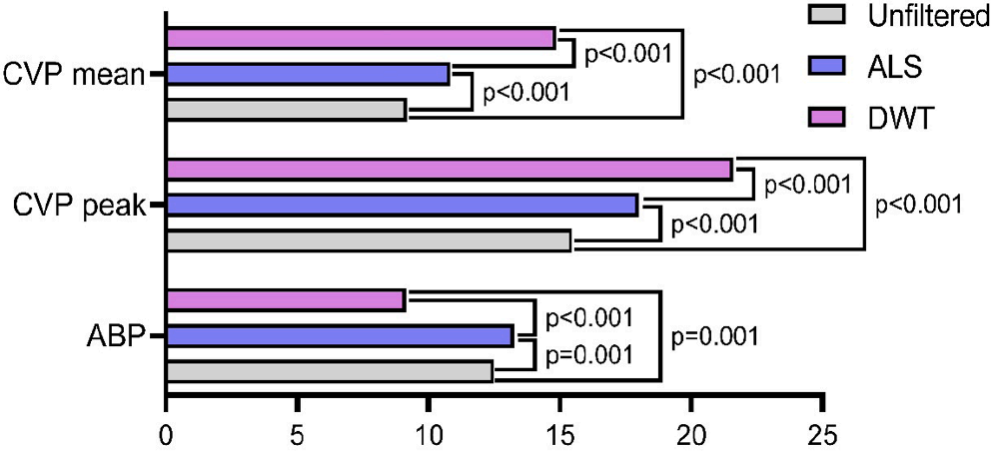
The impact of ALS and DWT filtering on the ABP, CVP peak and CVP mean values for AT window selection. The results represent a mean across all patients and all tested beats.

The results of filtering CVP and ABP signals are shown graphically in Appendix Fig. A4. Of note, DWT was the filtering method that maximised SNR for CVP signals, increasing the SNR for CVP peak and CVP mean by 40 % (P < 0.001) and 62 % respectively (P < 0.001) versus unfiltered signals. ALS filtering increased the signals by 16 % (P < 0.001) and 18 % (P < 0.001) for CVP peak/mean respectively.

In contrast, filtering ABP signals using DWT led to a decrease of 27 % (P < 0.001) in the SNR values versus unfiltered signals. In contrast, ALS filtering increased the SNR values by 6 % (P = 0.001) versus unfiltered.

Furthermore, the strongest negative correlation between individual ABP and CVP values occurred when using DWT to filter the CVP signal and leaving the ABP signal unfiltered (R = -0.59, p < 0.001, Table 1). Filtering the CVP with ALS did not improve the correlation.

### 3.3 Averaging window length

**Fig. 2:**
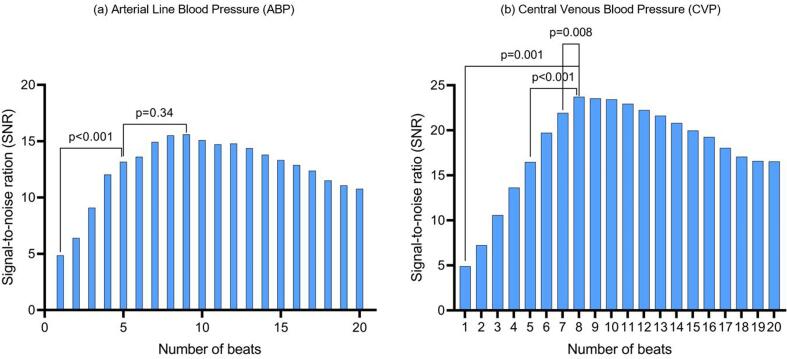
Change in mean SNR for ABP measurements (a) and CVP measurements (b) as progressively the averaging window length increases from 1 to 20 beats. (a) There was no significant improvement in SNR when the averaging window became longer than 5 heartbeats in length. (b) There was no increase in significance after 8 beats.

We first examined SNR for different fixed-length averaging windows ranging from 1 to 20 beats. The most efficient fixed averaging window for ABP was 5 beats, with a 19 % reduction in SNR observed when using a 20-beat window (P = 0.007) and a 67 % reduction with a single-beat window (P < 0.001). Although the SNR value peaked at a 9-beat window, there was no significant improvement in SNR after 5 beats (Fig. 2a).

The CVP SNR peaked later at 8 beats, with with a drop of 79 % in SNR observed when using a 1-beat window (P = 0.001), and a reduction of 17 % when using a 20-beat window (P = 0.07) (Fig. 2b). In contrast to ABP, 8 beats was the first point at which there was no subsequent significant improvement, and hence appears the most efficient average window length.

**Fig. 3:**
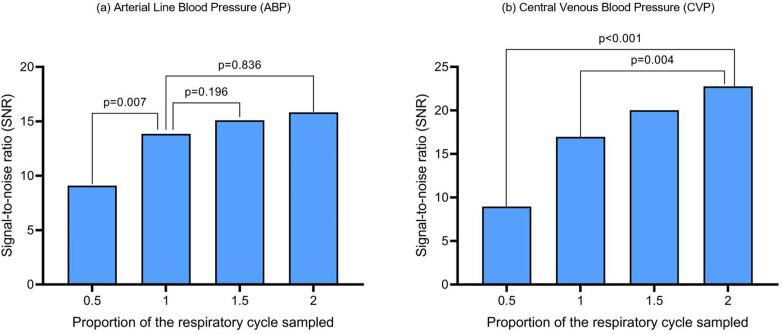
The effect of different respiratory cycle length proportions on the SNR values of ABP measurements (a) and CVP measurements (b) for an around-transition window using filtered signals. The results show a mean across all patients.

With regards to aligning beats to the respiratory cycle, for ABP the highest SNR was achieved when the number of beats equated to one respiratory cycle, with no significant improvement in SNR above one cycle (Fig. 3a). In contrast, for CVP peak measurements, there was a significant improvement in SNR when two respiratory cycles were used compared to one cycle (p = 0.004, Fig. 3b).

### 3.4 Correlation between ABP and CVP signals

As alluded to previously, there was a significant negative correlation between individual values of ABP and CVP when using CVP peak rather than mean, and this was strongest when CVP was filtered using DWT (Table 1). Furthermore, the highest SNR occurred when using an averaging window of 1 respiratory cycle for ABP and 2 respiratory cycles for CVP.

Thus we can combined these methods to examine the relationship between the optimal AV delay calculated using ABP and the optimal AV delay calculated using CVP under the following conditions: (a) CVP peak values are used, (b) CVP values are filtered using DWT, (c) 1 respiratory cycle is used for ABP, and (d) 2 respiratory cycles are used for CVP.

**Fig. A5:**
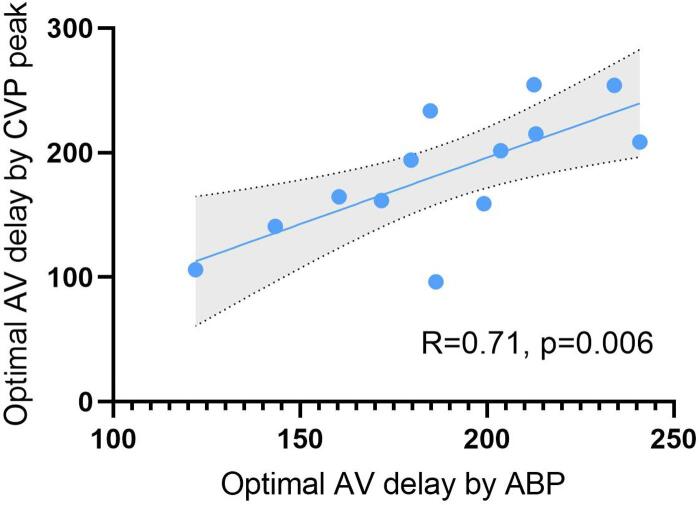
The relationship between the optima calculated using ABP and CVP measurements for a window length of one respiratory cycle for ABP and 2 respiratory cycles for CVP, where CVP was corrected using DWT.

Under these conditions, there is a strong relationship between the predicted optimal AV delay calculated by both CVP and ABP (R = 0.71, p = 0.006, Appendix Fig. A5).

**Fig. A6:**
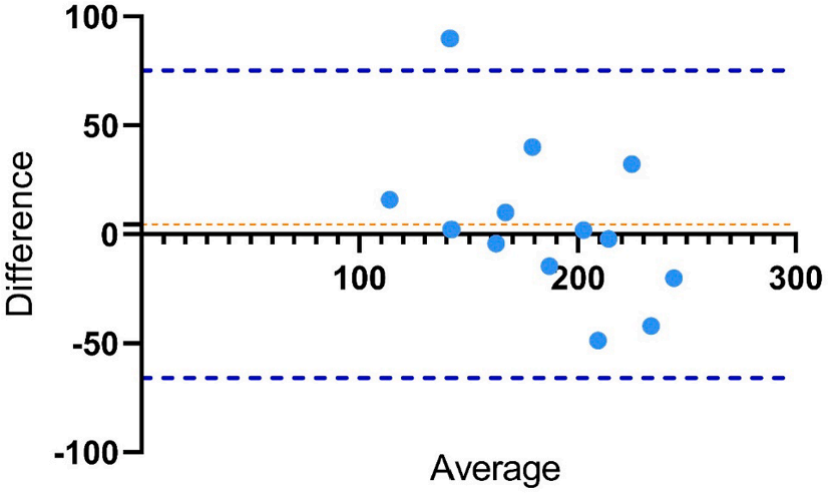
Bland-Altman plot between the ABP and CVP peak measurements. The dotted orange line shows the bias between the two methods, and the hashed blue lines show the upper and lower 95 % limits of agreement.

In contrast, if we violate these assumptions and use one respiratory cycle length for CVP and ABP, then the relationship weakens substantially (R = 0.50, p = 0.07). Furthermore, the agreement between the ABP and CVP-calculated optima does not seem to have any systematic bias as the mean values change (Appendix Fig. A6).

## 4 Discussion

This study has shown that different methods of data processing can improve the quality of data when using CVP as a method of optimising AV delay in temporary pacemakers after cardiac surgery. Firstly, to maximise signal-to-noise ratio, data around the transition should be analysed rather than data solely taken from after the transition. Secondly, the optimal window length is different for ABP and CVP analysis, where analysing a single respiratory cycle length is adequate for ABP but two cycles are optimal for CVP. Finally, respiratory artefact correction can be further augmented by using Discrete Wavelet Transform to filter CVP signals.

Analysing data in the post-transition has theoretical advantages. When moving from a haemodynamically more efficient AVD to to a less efficient AVD (for example transitioning from an AVD of 120 ms to 40 ms) the cardiac output is likely to drop. When looking at this from an arterial perspective we see this in the first five beats after the transition, and the analysis of beats 6 to 20 adds little value. This is because the patient's homeostatic mechanisms are initiated to maintain constant perfusion to vital organs, largely due to vasoconstriction in this immediate time-frame. Therefore, in theory, analysing beats only after the transition could see a greater signal.

In practice, however, whilst the signal was marginally higher, the noise was substantially greater as half the data is discarded using this method, and biological systems are fundamentally noisy. Therefore, the SNR was significantly better when analysing beats around the transition rather than post-transition only. This effect was maintained when examining both ABP and CVP data, where using a post-transition window reduced the SNR by around 20 % for both.

In contrast, optimal window length was different when examining both CVP and ABP data. We found that whilst one respiratory cycle was adequate for correcting ABP signals, using two respiratory cycles maximised the signal-to-noise ratio of CVP signals.

In this case we have two competing effects. On one hand, as discussed above, the signals around the transition contain the highest proportion of signal. On the other hand, respiratory variation adds more noise to the system. CVP signals fluctuate more with respiration [9], in proportional terms, than ABP signals. Analysing the CVP signals over two respiratory cycles reduced the noise more than it reduced the signal by including less data-rich heartbeats further from the transition point, resulting in a higher SNR.

Conversely, analysing one respiratory cycle for ABP was most efficient, showing that the contest between higher signals around the transition versus noise reduction from signal-averaging over more respiratory cycles was in favour of the former. This probably explains why a higher fixed number of beats was most efficient for CVP compared to ABP (8 versus 5 beats). Therefore, when calculating the optimal AV delay using CVP, 2 respiratory cycles results in a higher SNR than one cycle, but when using ABP one cycle is sufficient.

Finally, further respiratory correction can be applied using mathematical techniques. Using Discrete Wavelet Transform made a large difference to the SNR of CVP signals, increasing the SNR by 62 %. The effect of filtering was much less pronounced for ABP signals, where the best filtering method (ALS) increased the SNR by only 6 %, although this was still a significant increase (p = 0.001, Fig. 1). DWT had a negative effect on ABP SNR ratios, and therefore this study suggests that ALS is more appropriate for ABP baseline correction.

When we combine the methods for SNR maximisation into a single protocol, the resultant optimal AVD as calculated by either CVP or ABP showed very good agreement (Appendix Fig. A5 and Fig. A6) with R values rising from 0.5 to 0.71. This includes all data from all patients, even those where there was significantly noisy data. Unpublished work from our group has showed that the initiation of a two-step quality control algorithm before analysis further increases the strength of this relationship: the combination of the techniques reported in this paper to data that has passed quality control is another future avenue for research. It also shows that CVP data requires more careful processing that ABP data, which possibly is why the CVP has not been used for AVD optimisation before.

Our previous work and this study have shown that CVP could be used as a target for optimising AV delay in temporary dual-chamber pacing. The gains in efficiency are important if these algorithms are included in implanted devices because they decrease then number of replicates and signal analysis required by the device, with implications for prolonging battery life. This would lead to fewer generator replacements, where each subsequent generator replacement at the same site doubles the risk of infection [12].

This pilot study is limited by its use of patients with temporary pacing as study subjects: in future examination of CVP in patients with cardiac resynchronisation therapy may be useful in assessing the generalisability of this method.

## 5 Conclusion and future directions

This study has shown that CVP can be used to optimise AVD in patients requiring temporary pacing after surgery, but that the analysis methods of recorded CVP data can have large effects on the signal-to-noise ratio. More specifically, analysing two respiratory cycle lengths of heartbeats around the transition is optimal, and filtering with DWT the most effective form of baseline correction. Assimilating thee into a single protocol results in the highest agreement between CVP-calculated optima and single respiratory-cycle ABP-calculated optima.

If CVP analysis becomes integrated into implanted devices then these methods of more efficient signal correction will be vital in improving device performance and longevity.

## CRediT authorship contribution statement

**Ioana Cretu:** Writing – review & editing, Writing – original draft, Visualization, Validation, Software, Resources, Project administration, Methodology, Investigation, Formal analysis, Data curation, Conceptualization. **Alexander Tindale:** Writing – review & editing, Writing – original draft, Visualization, Validation, Methodology, Investigation, Formal analysis, Data curation, Conceptualization. **Maysam Abbod:** Supervision, Funding acquisition. **Wamadeva Balachandran:** Supervision, Funding acquisition. **Ashraf W. Khir:** Supervision, Funding acquisition. **Hongying Meng:** Writing – review & editing, Supervision, Resources, Project administration, Funding acquisition, Formal analysis, Conceptualization.

## Declaration of competing interest

The authors declare that they have no known competing financial interests or personal relationships that could have appeared to influence the work reported in this paper.
